# Factors Influencing Adolescents' Dietary Behaviors in the School and Home Environment in Addis Ababa, Ethiopia

**DOI:** 10.3389/fpubh.2022.861463

**Published:** 2022-04-08

**Authors:** Ursula Trübswasser, Elise F. Talsma, Selamawit Ekubay, Maartje P. Poelman, Michelle Holdsworth, Edith J. M. Feskens, Kaleab Baye

**Affiliations:** ^1^Division of Human Nutrition and Health, Wageningen University, Wageningen, Netherlands; ^2^Center for Food Science and Nutrition, Addis Ababa University, Addis Ababa, Ethiopia; ^3^Department of Social Sciences, Chair Group Consumption and Healthy Lifestyles, Wageningen University, Wageningen, Netherlands; ^4^UMR MoISA (Montpellier Interdisciplinary Centre on Sustainable Agri-Food Systems), (Univ Montpellier, CIRAD, CIHEAM-IAMM, INRAE, Institut Agro, IRD), Montpellier, France

**Keywords:** food environment, urban, food advertising, food outlet, adolescents

## Abstract

**Background:**

Malnutrition affects many adolescents in Ethiopia. Over one-third of adolescent girls and two-thirds of boys are thin. Overweight and obesity in Ethiopia is mostly a concern in urban populations of higher wealth quintiles. Urbanization and globalization of diets is shifting food environments. The objective of this study was to assess whether food environments in and around schools in urban Ethiopia influence dietary diversity, quality, BMI status or perceptions of adolescents.

**Methods:**

Twelve high schools were selected in Addis Ababa (private/government). From each school, 20 pupils aged 15–19 years were randomly selected (*n* = 217) and interviewed about assets in their households, their diets (categorized into 10 food groups of the Minimum Dietary Diversity, the Global Dietary Recommendations scores and four categories of the NOVA classification based on level of processing) and their use of pocket money. In addition, food environment audits were conducted within the school compound and a 0.5 km radius around each school and types of food outlets.

**Results:**

On average there were 436 food outlets and 246 food or drink advertisements around each school. The majority of the advertisements (89.9%) were of ultra-processed foods, mostly sugar-sweetened beverages (SSBs). Most were positioned on food outlets (89.1%). SSBs or sweets were visibly on display in 26.3% of the outlets and fresh fruits and vegetables in 17.9% of outlets. Dietary diversity of adolescents was poor with an average of 3.6 food groups out of 10 consumed in the last 24 h. Ultra-processed foods and beverages were consumed by 23.5% of adolescents. The majority of adolescents spent their pocket money on SSBs, sweets or fried foods. Our analysis found that higher assets in adolescents' households were associated with higher dietary diversity and consumption of healthy food groups. We found no association between the food environment and dietary indicators or the BMI-z-score.

**Conclusion:**

While the school food environments investigated were not conducive with promoting healthy dietary behaviors, we cannot conclude that these environmental factors directly influence adolescents' diets. The pervasive advertising and availability of unhealthy foods and beverages requires policy action for healthy school food environments.

## Introduction

Dietary behaviors within populations are highly dependent on which food and beverages are available, affordable, safe or convenient in their surroundings (food environment) (1). Food environments can be defined as the spaces where individuals interact with the food system and encompass availability, promotion, quality, convenience and physical and economic access (2). Availability and cost influence adolescents' dietary behaviors, as well as their appeal and aspirational association (3). Food environments are changing globally due to expanding urbanization, technology, trade and labor markets. These changes are leading to increased availability of energy-dense, nutrient-poor, ultra-processed foods and beverages associated with the ‘nutrition transition' (4). This in turn can negatively affect dietary quality, with high consumption of ultra-processed foods and beverages, such as sugar-sweetened beverages (SSBs). SSBs are associated with poor nutrition and health outcomes, including overweight and obesity and diet-related non-communicable diseases (5).

The nutrition transition has taken place in high-income countries over the last few decades and is well underway in low- and middle-income countries (LMICs) (5, 6), including in Africa (7).

Changing food environments can influence the dietary habits of adolescents (8), who spend a lot of their time at school and are at a critical time of habit formation and increasing autonomy (9–11). The promotion of ultra-processed foods and beverages often targets children and adolescents to generate brand awareness, preference and loyalty, securing a future consumer base (12). Hence, food environments around schools can play a critical role in adolescents' diets (9, 13, 14). As studies from mostly high-income countries but also LMICs have shown, the availability of unhealthy food or beverages inside or around schools, in the absence of parental supervision, negatively affects dietary choices (9, 15–18). In LMICs, parental education and occupation were also found to be associated with better nutritional status and dietary behaviors (19, 20).

Adolescents' diets in LMICs seem to be inadequate: predominantly cereal-based and limited in terms of animal-source foods, fruit and vegetables (21). Particularly in urban areas, increased consumption of processed energy-dense and nutrient-poor foods and drinks has been reported (8). In Ethiopia, up to a third of adolescents consume SSBs on a daily basis (22). Whether these dietary behaviors are related to the food environment surrounding schools in Ethiopia remains unknown. Pupils in many countries, including Ethiopia, have limited pocket money to spend and their caregivers act as “gatekeepers” of their choices (10). However, how Ethiopian adolescents from different socio-economic backgrounds are exposed to food outlets on the way to and from school and how this can affect their dietary and purchasing behaviors and nutritional status requires exploration. Therefore, this study assessed whether food environments in and around schools in urban Ethiopia influence dietary diversity, quality, body mass index (BMI) status or adolescents' perceptions of their school and home food environment.

## Methods

### Study Design and Context

A cross-sectional study was conducted, including school food environment audits as well as interviews with students of private and government schools. The selection of private and government schools was used as a proxy for socio-economic status, based on the rationale that private schools charge tuition fees (23). The schools were selected in collaboration with Addis Ababa Bureau of Education and the Addis Ababa sub-city administration using a list of all middle and high schools in the city. Our aim was to identify a pair of one private and one government school that had less than a 0.5 km distance between them to ensure that participants from both schools shared the same food environment. Twelve schools fulfilled this criterion and were located in six different sub-cities of Addis Ababa, Ethiopia (Arada, Bole, Kality, Kirkos, Kolfe-Keranio and Laphto).

### Participant Recruitment

Given the scarcity of data on adolescents in schools, and the multiple outcomes of interest, the sample size was calculated to detect a medium effect size (Cohen's *d*; 0.5 SD) difference between two means, assuming α = 0.05 and power = 0.95. Lists of all enrolled students and their ages were obtained for all the sampled schools. From these lists, containing a total of 1,500 students/school on average, 20 adolescents (aged 15–19 years) were randomly selected. In every school, a teacher assisted with identifying adolescents and informing them and their parents about the study. This resulted in a total target sample of 240 adolescents who were invited for interview.

### Data Collection Procedure

Enumerators with experience in data collection and with an excellent command of the local language, Amharic, were recruited and trained for 3 days on interviewing skills, dietary intake assessments and anthropometric measurements. The training was followed by a pre-test of the adolescents' questionnaire and the food environment audit tools, which permitted adaptations when necessary. Using interview-administered questionnaires, information on socio-demographic characteristics and food consumption was assessed for the recruited adolescent participants in all schools. Moreover, height and weight were measured to assess the BMI status. As a next step, the external and internal food environment in and around all 12 schools was assessed using protocols from the International Network for Food and Obesity/Non-Communicable Diseases Research, Monitoring and Action Support (INFORMAS) network to measure food environments that contain elements on food promotion (advertising) and types of food outlets (24). Data were collected between March and June 2019.

### Socio-Economic Variables and Purchasing Behavior

Since adolescents were not able to provide information on family income, they were asked about 13 different assets that their family owned, based on the family affluence scale (25) and the asset list included in the Ethiopian Demographic Health Survey (26) (yes = 1, no = 0). From the responses, we calculated the sum of all scores by assigning one point to each asset (min–max score = 0–13). Additionally, we asked if adolescents had their own bedroom, received pocket money and how they commuted to school. If they received pocket money, we asked for the amount they received per week and if they spent it on SSBs, sweets, fruit or fried foods.

### Adolescents' Perceptions of Their School and Home Food Environment

We assessed adolescents' perceptions in terms of the availability of fruit and vegetables or snacks at their homes, as well as their perception of availability and advertising of (un)healthy foods in the school food environment. We used tested statements previously used in studies with adolescents (20) or in studies assessing perceptions of the food environment (27, 28). The statements were read to the participants and they reported their agreement with each statement using a five-point Likert scale (1 = *strongly disagree* to 5 = *strongly agree*).

### Dietary Intake Assessment

Interviewers used an open-ended qualitative 24-h recall, starting with an unstructured listing of all foods and beverages consumed, followed by memory cues to assess consumption over the previous 24 h.

### Anthropometric Measures

Height and weight were measured with standardized measurements in triplicate. Height was measured with stadiometers (SECA 213) in a standing position without shoes and was recorded to the nearest 0.1 cm (29). Electronic weighing scales (SECA 872) with a weighing capacity of 10–140 kg were used to assess the weight of all participants to the nearest 0.1 kg.

### Food Environment Assessment

For each school, the external food environment around the school within a radius of 0.5 km was assessed for visibility and advertising of foods and beverages using the INFORMAS protocol for “Promotion – Outdoor Advertising,” which has been used in other LMICs (24). Food advertisements were categorized as advertisements promoting food or beverage brands on stationary objects, such as posters, banners, bus-stop advertisements, flags, furniture, umbrellas, tables, fridges or free-standing signs in public spaces. For every advertisement, the category, location (GPS code), size (small, medium or large) and type of food or beverage advertised were recorded. Food outlets were assessed in terms of outlet categories, location (GIS code), presence of advertising and display of fruit, vegetables or SSBs. Enumerators did not enter any stores but walked up and down every single street in the defined radius.

The data collection tool for the food environment assessment was pre-tested in October 2018, in central Addis Ababa (Arat Kilo), in an area close to two of the schools. During the pre-test 12 categories of food outlets were identified, as any shop, café or restaurant selling food or beverages, and categorized as “informal” if the shop's structure (if any) was movable and not permanent (see Supplementary Material 1 for the 12 categories). The tool was tested on each food outlet type and the findings were used to amend the tool. The food environment of the first two schools was assessed by two independent teams of enumerators to align the data collection procedure and assess inter-rater reliability. Within the school compound, we assessed any presence of food or beverage advertising and whether SSBs were sold at the school cafeteria.

### Data Quality Control

All tools were translated into Amharic, a local language. The quality of the translation was checked by back-translating the questionnaires into English. All data from the individual interviews and the food environment were entered on tablets (Lenovo TAB 7 essentials) and questionnaires were programmed with Skip Logic using the Open Data Kit, which is an electronic data collection program. Data were uploaded daily on a secure, centrally managed server, allowing daily quality checks from the first author. Daily debriefs with enumerators were conducted by the first and third authors to discuss and resolve any potential challenges.

### Data Analysis

All consumed food items and beverages from the previous 24 h were categorized into 10 food groups following the Minimum Dietary Diversity for Women (MDD-W) approach, which is useful to reflect the micronutrient adequacy of diets and is recommended for use in LMICs (30). In addition, foods and beverages were assigned to one of four categories of the NOVA classification based on their level of processing (31). However, our study only focused on whether the foods and beverages consumed fell into the fourth NOVA category of ultra-processed foods. Dietary data were also categorized into Global Dietary Recommendations (GDR) scores, which in addition to the MDD-W add value as indicators of dietary quality. Diet patterns were assessed in terms of their adherence to global dietary recommendations for fruit and vegetables, dietary fiber, free sugars, saturated fat, total fat, legumes, nuts and seeds, whole grains and processed meats. The GDR score is composed of two subcomponents: GDR-Healthy, which is an indicator of the recommendations on nine groups of “healthy” foods; and GDR-Limit, which is an indicator of the recommendations on eight dietary components to limit, such as snacks, ultra-processed foods/beverages and deep-fried foods (32).

Dietary data are presented in terms of mean dietary diversity scores based on the number of food groups (min–max score = 0–10), the mean GDR-Healthy, GDR-Limit and GDR total (calculated by subtracting GDR-Limit from GDR-Healthy and adding 9 to transform the indicator to a range of 0–18), the percentage of adolescents consuming different food groups and ultra-processed foods (based on the NOVA classification) and the percentage of adolescents.

The BMI-for-age *z*-scores were calculated using WHO AnthroPlus v 1.0.4 to assess the nutritional status of the participants.

IBM SPSS Statistics v25.0 was used for data analysis. Continuous variables are presented as mean ± SD and counts as frequency (percentage). To estimate the relationship of food environment and socio-economic indicators with dietary and nutritional outcomes, we performed a multiple linear regression analysis with dietary diversity scores, diet quality (GDR-Healthy, GDR-Limit) scores or BMI *z*-scores as the dependent variable and number of outlets around the school, SSB advertising or sale within the school compound, number of assets in the household and pocket money of the student as independent variables. Education level of the parents was included in the model as a potential confounding factor. We dichotomized the food environment variables (number of outlets) into low density (defined as equal or below the median) or high density (values above the median) so that the estimated coefficient was not influenced by outliers.

Perceptions of the school and home food environment were also dichotomized by collapsing “strongly agree and agree” together and “strongly disagree and disagree” together. We then performed a binary logistic regression of the perception variables with the same food environment and socio-economic variables as independent variables. Statistical significance was set at α = 0.05 and all tests were two-sided.

## Results

### Description of Sample

From a total of 240 eligible adolescents, 217 completed the study; the average age of participants was 17.2 (SD 1.0) years and slightly more than half (59%) were female (Table 1). More adolescents from private schools received pocket money and the amounts were also higher for private school students. Over three-quarters of adolescents (79%) walked <10 min from a car or bus to the school gate. Only private school children reported traveling to school in their parent's car (data not shown).

**Table 1:** Socio-demographic and anthropometric characteristics of study participants (total and separated by school type).

	**Total** **(*n* = 217)**	**Private schools** **(*n* = 107)**	**Government** **schools ** **(*n* = 110)**	***P* value**
**Mean** **±SD or** ***n (%)***				
Age (years)	17.2 ± 1.0	17.1 ± 1.1	17.2 ± 0.9	0.25
**Gender**				
Female	128 (59)	59 (55.1)	69 (62.7)	0.27
**Socio-economic indicators**				
Number of assets^2^	10.1 ± 1.6	10.8 ± 1.4	10.8 ± 1.4	<0.001
Own bedroom	88 (40.6)	63 (58.9)	25 (22.7)	<0.001
Receives pocket money	161 (74.2)	93 (43.3)	68 (31.6)	<0.001
Weekly pocket money amount (Ethiopian Birr)^1^	94.8 ± 87.2	110.9 ± 101.0	72.9 ± 57.4	<0.001
**Nutritional status**				
BMI-for-age *z*-score (mean)	−0.7 ± 1.2	−0.6 ± 1.3	−0.8 ± 1.1	0.15
Underweight^3^	28 (12.9)	16 (14.9)	12 (10.9)	0.21
Overweight^3^	16 (7.4)	11(10.3)	5 (4.6)	0.21
Obesity^3^	4 (1.8)	2 (1.9)	2 (1.8)	0.21
Normal weight	168 (77.8)	78 (72.9)	90 (82.6)	0.21

*^1^Ethiopian currency*.

*^2^Min–max score = 0–13*.

*^3^Underweight, z-score < −2; overweight, z-score > +1 and < +2; obese, z-score > +2*.

### BMI, Dietary Diversity and Quality of Adolescents

Over three-quarters of adolescents (77%) had a normal weight, whereas 13% were classified as underweight and 9% as overweight or obese (Table 1); the mean BMI *z*-score was −0.7 (SD 1.2). The mean dietary diversity (DD) score of adolescents was 3.6 (SD 0.9) out of 10 food groups (Table 2). Adolescents from private schools had significantly higher mean DD than their peers from government schools (*P* < 0.05). With regard to the GDR, on average, adolescents consumed 3.4 out of the 9 health-promoting food groups (GDR-Healthy) and <1 food or drink of the 8 groups that should be limited or avoided (GDR-Limit). Private school adolescents had higher GDR-Limit scores. In the 24-h period before the interview, most adolescents consumed grains (99%), vegetables (mostly onions: 98%) and pulses (77%), but eggs (3%), dairy foods (5%) or nuts (6%) were rarely consumed. Dark-green leafy vegetables and other vitamin A-rich fruit or vegetables were consumed by less than one-third of adolescents. In contrast, ultra-processed foods and beverages, basically sweets and SSBs, were consumed by almost a quarter (23.5%) of adolescents. Meat consumption was higher in private school adolescents (24.3 vs. 6.4% in government schools).

**Table 2:** Dietary and purchasing behavior of study participants: consumption and purchase by food group and level of processing (total and separated by school type).

	**All schools (*n* = 217)**	**Private** **(*n* = 107)**	**Government (*n* = 110)**	***P* value**
**Mean** **±SD or** ***n (%)***				
**Dietary diversity**				
Mean dietary diversity score	3.6 ± 0.9	3.7 ± 1.0	3.4 ± 0.8	<0.001
**GDR**				
GDR Total GDR-Healthy GDR-Limit	11.72 ±1.26 3.35 ± 1.03 0.64 ± 0.75	11.64 ± 1.36 3.34 ± 1.14 0.79 ± 0.79	11.80 ± 1.16 3.28 ± 0.90 0.48 ± 0.67	0.34 0.29 <0.001
**Consumption of different food groups**				
Grain	216 (99.5)	107 (100)	109 (99.1)	1.00
Pulses	168 (77.4)	82 (76.6)	86 (78.2)	0.87
Nuts	14 (6.5)	10 (9.3)	4 (3.6)	0.10
Dairy	11 (5.1)	6 (5/6)	5 (4.5)	0.77
Meat	33 (15.2)	26 (24.3)	7 (6.4)	<0.001
Egg	6 (2.8)	5 (4.7)	1 (0.9)	0.12
Dark-green leafy vegetables	44 (20.3)	27 (25.2)	17 (15.5)	0.09
Vitamin A-rich fruit or vegetables	56 (25.8)	27 (25.2)	29 (26.4)	0.88
Other vegetables	213 (98.2)	105 (98.1)	108 (98.2)	1.00
Other fruit	11 (5.1)	5 (4.7)	6 (5.5)	1.00
Ultra-processed foods or beverages	51 (23.5)	29 (27.1)	22 (20)	0.26
**Use of pocket money for** ^**1**^				
SSBs Sweets Fruit Fried food	31 (19.3) 40 (24.8) 9(5.6) 89 (55.3)	25 (26.9) 25 (26.9) 6 (6.5) 57 (61.3)	6 (8.8) 15 (22.1) 3 (4.4) 32 (47.0)	0.01 0.84 1.00 0.20

*^1^n = 161: n = 93 for private schools; n, 68 for government schools; GDR, Global Dietary Recommendations; SSBs, sugar-sweetened beverages*.

Three-quarters of adolescents (74%) received pocket money, which they spent on fried food (55%), sweets (25%) or SSBs (19%). While this was the case for all adolescents receiving pocket money, private school attendance was associated with purchasing more SSBs.

### Adolescents' Perceptions of Their Home and School Food Environment

Adolescents from both schools agreed that food outlets around the school sell snack foods, although they also perceived healthy food to be available (Table 3). While most of them perceived the advertising to be of unhealthy foods or beverages, most also disagreed that there was a lot of advertising in the neighborhood. Having fruit and vegetables available in their homes, in addition to unhealthy snacks, was more likely to be reported by government school adolescents.

**Table 3:** Adolescents' perception of the school and home food environment.

**Statement**	**All schools ** **(*n* = 217)**	**Private** ** (*n* = 107)**	**Government** ** (*n* = 110)**	***P* value**
***n (%)***				
**In my house we always have fruit and vegetables**				
(Strongly) disagree (Strongly) agree Neither agree nor disagree	105(48.4) 105 (48.4) 7 (3.2)	35 (32.7) 63 (58.9) 9 (8.4)	68 (64.8) 33 (31.4) 9 (8.2)	<0.001
**In my house we always have fast food, sodas and snacks**				
(Strongly) disagree (Strongly) agree Neither agree nor disagree	174(80.2) 30 (13.8) 13 (6.0)	76 (71.0) 23 (21.5) 8 (7.5)	98(89.1) 7 (6.4) 5 (4.5)	<0.001
**There are lots of shops selling snack food in the school neighborhood**				
(Strongly) disagree (Strongly) agree Neither agree nor disagree	29 (13.4) 184 (84.8) 4 (1.8)	16 (15.0) 89 (83.2) 2 (1.9)	13 (11.8) 95 (86.4) 2 (1.8)	0.79
**Healthy foods are available in the school neighborhood**				
(Strongly) disagree (Strongly) agree Neither agree nor disagree	27 (12.4) 176 (81.1) 14 (6.5)	12 (11.2) 90 (84.1) 5 (4.7)	15 (13.6) 86 (78.2) 9 (8.2)	0.47
**There is a lot of food advertising in the school neighborhood**				
(Strongly) disagree (Strongly) agree Neither agree nor disagree	127 (58.5) 84 (38.7) 6 (2.8)	63 (58.9) 41 (38.3) 3 (2.8)	64 (58.2) 43 (39.1) 3 (2.7)	0.99
**The advertising is mostly promoting unhealthy food and drink**				
(Strongly) disagree (Strongly) agree Neither agree nor disagree	77 (35.5) 127 (58.5) 13 (6.0)	36 (33.6) 65 (60.7) 6 (5.6)	41 (37.3) 63 (56.4) 7 (6.4)	0.81

### Description of the Internal and External Food Environment

Within the school compound, we found that all but two private schools sold SSBs at their cafeteria and three government schools had advertising for SSBs on the school compound. In the 0.5 km radius around a private or a public school, we found an average of 436 (SD 366) food outlets, but with large differences between sub-cities, ranging from 113 to 924 food outlets. The schools in the Kality and Arada sub-cities had the highest numbers of food outlets surrounding them (Table 4), which is due to the dense inner-city location of Arada and the large market area in Kality. Consequently, the absolute exposure to outlets selling fruit and vegetables was highest in Kality. Display of SSBs was highest in food outlets in Arada. Kiosks were the most common food outlets, representing 21.9% of all outlets in all clusters, and they had the largest proportion of advertisement and displays of SSBs (46.9% and 60.0%, respectively). The absolute number of advertisements was also highest in Arada (*n* = 720) and Kality (*n* = 405). However, in all sub-cities most of these advertisements promoted SSBs (89.9%). Most advertisements were positioned on food outlets (89.1%) and presented as posters, boards or banners. The second most common form of advertising was as part of the food outlet's equipment, such as umbrellas, tablecloths or fridges (20.0%). The least common forms of advertisements were large billboards (0.9%).

**Table 4:** Food outlets (type, characteristics) and advertising in and around (0.5 km radius) schools (private and government) in the respective sub-city of Addis Ababa, Ethiopia, *n* (%).

	**Mean, all sub-cities**	**Arada**		**Bole**		**Kality**		**Kirkos**		**Kolfe-Keranio**		**Laphto**	
**School** ^**1**^		PS	GS	PS	GS	PS	GS	PS	GS	PS	GS	PS	GS
SSBs sold at school		y	y	n	y	y	y	y	y	y	y	n	y
SSBs advertised at school		n	y	n	n	n	n	n	n	n	y	n	y
**Type of outlet**														
Outlets, total	436	832		134		924		155		460		113	
Kiosks	93	121 (14.5)		26 (19.4)		193 (20.9)		46 (29.7)		136 (29.6)		38 (33.6)	
Supermarkets	5	9 (1.1)		3 (2.2)		4 (0.5)		0		4 (0.9)		9 (7.9)	
Sweet seller, informal	35	100 (12.0)		7 (5.2)		39 (4.2)		0		63 (13.7)		0	
Fruit and vegetable stall	39	34 (4.1)		7 (5.2)		156 (17.2)		8 (5.2)		23 (5.0)		4 (3.5)	
Local café	101	313 (37.6)		38 (28.4)		116 (12.5)		51 (32.9)		70 (15.2)		18 (15.9)	
Other	163	255 (30.6)		53 (39.6)		416 (45.0)		50 (32.3)		164 (35.7)		44 (38.9)	
**Outlets with food or beverage visibility/advertising**														
FV visibly displayed in outlet	78	28 (3.4)		21 (15.7)		295 (31.9)		19 (12.3)		85 (18.5)		21 (18.6)	
SSBs visibly displayed in outlet	115	353 (42.4)		82 (61.2)		145 (15.7)		41 (26.5)		48 (10.4)		20 (17.7)	
Food and beverage advertising on outlet	103	234 (28.1)		41 (30.6)		195 (21.1)		48 (30.9)		74 (16.1)		25 (22.1)	
**Food and beverage advertising**														
Advertising, total	246	720		87		405		99		126		44	
Advertising of ultra-processed food or beverages	222	628 (87.2)		77 (88.5)		388 (95.8)		92 (92.9)		113 (89.7)		34 (77.3)	
Position of advertising on food outlet	220	648 (90.0)		80 (92.0)		359 (88.6)		90 (90.9)		105 (83.3)		40 (90.9)	
Advertising type (poster, board or banner)	176	401 (55.7)		74 (85.0)		359 (88.6)		91 (91.9)		89 (70.7)		33 (88.6)	

*^1^PS, private school; GS, government school; y, yes; n, no; FV, fruits or vegetables; SSBs, sugar-sweetened beverages*.

### Factors Influencing Adolescents' Diets and BMI Status

Dietary diversity was higher in adolescents with assets in the household when considering both food environment and socio-economic variables; this was also the case for the GDR-Healthy score (Table 5). This association remained when including parents' education into the model. No other associations were found with consumption of unhealthy food groups (GDR-Limit) or BMI-for-age and socio-economic indicators. Factors in the food environment were neither associated with dietary scores nor BMI-for age *z*-scores.

**Table 5:** Potential influencing factors on dietary diversity, quality or nutritional status by applying multiple linear regression.

	**Dietary diversity**			**GDR-Healthy**			**GDR-Limit**			**BMI-for-age**		
**Predictors**	**Beta**	**S.E**.	***P***	**Beta**	**S.E**.	***P***	**Beta**	**S.E**.	***P***	**Beta**	**S.E**.	***P***
**Food environment**												
High number of food outlets (> 460)	−0.18	0.16	0.25	−0.11	0.17	0.52	0.04	0.12	0.73	0.14	0.19	0.48
SSBs sold at school	0.09	0.21	0.67	0.21	0.23	0.36	0.25	0.16	0.14	0.43	0.26	0.09
SSBs advertised at school	0.07	0.17	0.67	−0.98	0.18	0.59	0.04	0.13	0.76	0.16	0.21	0.45
**Socio-economic**												
Asset score	0.11	0.05	0.04	0.12	0.06	0.03	0.02	0.04	0.65	0.09	0.06	0.15
Receiving pocket money	−0.83	0.18	0.64	0.23	0.19	0.23	0.13	0.14	0.37	0.22	0.22	0.31
**Education of parents**	−0.04	0.08	0.63	0.01	0.09	0.94	0.12	0.06	0.06	0.01	0.10	0.96

*S.E., standard error; GDR, Global Dietary Recommendations; BMI, body mass index; SSBs, sugar-sweetened beverages*.

Adolescents' perceptions of the home environment were also associated with assets and pocket money (data not shown). Adolescents from households with more assets or pocket money were more likely to perceive that, at their homes, they always had fruit and vegetables as well as snacks, which could be an explanation for the positive association of assets with dietary outcomes.

## Discussion

The aim of our study was to examine food environments in and around schools in urban Ethiopia and to explore how they might influence dietary diversity, quality, BMI status or adolescents' perceptions of their school and home food environment. We observed that high dietary diversity as well as higher consumption of healthy foods was associated with adolescents from households with more assets. For both groups of students, our study found a high density of food outlets within the 0.5 km radius around the schools, as well as widespread promotion and display of ultra-processed foods and beverages in and around the schools. While such an environment is not conducive to promoting healthy dietary behaviors, we cannot conclude that these environmental factors directly explain adolescents' diet or weight status.

The differences between private and government-school adolescents in terms of their dietary diversity and purchasing of SSBs could be explained by the socio-economic status of their families, which we assessed using the number of assets or the amount of pocket money the adolescents receive. Parents who give pocket money without spending stipulations could create financial autonomy, but the lack of supervision could also potentially worsen the unhealthy dietary behaviors of adolescents (33). Studies have shown the different roles that parents play in food consumption. Mothers preparing food at home have been described as a positive influence, whereas high-income parents who are too busy to prepare food may become negative role models (33–35). In our sample, adolescents from households with more assets also perceived that both healthy and unhealthy foods were available in their households. Globally, consuming SSBs is socially stratified, with high-income groups consuming them in LMICs and shifting to lower income groups as a country's income level increases (5). Our data support this, as purchases of SSBs in Ethiopia were greater among participants with more household assets.

We observed that adolescents who spent their pocket money on food/beverages were more likely to spend it on fried foods, sweets or SSBs rather than on fruit. Purchasing little or no fruit on the way to or from school, even though it is widely available, could also be due to food safety concerns related to fruit sold in unhygienic conditions or lack of clean water to wash it (36). Furthermore, adolescents' purchasing choices provided an insight into their preference for fried food, sweets or SSBs over fruit. As opposed to fresh fruit, adolescents could consider packaged ultra-processed foods or beverages to be a safer and socially more acceptable and desirable option (33, 36).

Ultra-processed food and beverages, such as sweets and SSBs, were found to be widely advertised and displayed visibly in the food outlets surrounding schools. A recent review found that in high-income countries, unhealthy retail food establishments are increasing and tend to cluster around schools (16). Furthermore, in LMICs, food companies are developing extensive distribution networks, providing point-of-sale advertising materials or free distributions (5), and using spaces with the highest consumer traffic to tempt consumers into buying ultra-processed foods or beverages (37). Unlike other studies from LMICs, we did not find that unhealthy food environments around schools were directly linked with poorer dietary quality (9) or higher BMI (38, 39). This could largely be due to the fact that students take their own lunch to school, with their parents acting as “gatekeepers” of their choices (10). Therefore, parental and social norms could have a stronger influence on adolescents' diets than the physical food environment. However, adolescents are at a critical stage in life, learning to make their own dietary choices, and with decreasing influence of parents and increasing financial autonomy their dietary behaviors could be more strongly influenced by the food environment (10).

Schools provide a well-defined and preferred setting for prevention strategies to improve the diets of children and adolescents (14). The external and internal school environment assessed by our study was not conducive to healthy food choices. Current Ethiopian school policies are limited to school feeding and food safety, and lack actions on the availability or advertising of food in and around schools (40), which are needed to extend policy action to focus on addressing all forms of malnutrition.

### Strengths and Limitations

To our knowledge, this is the first study to map food environments in and around schools in urban Ethiopia and explore how this is associated with the dietary behaviors and weight status of adolescents. Due to its cross-sectional nature, the study only provides a snapshot of the prevailing food environment and diet diversity at the time of the survey and therefore does not allow causal inferences to be made. Assessing only the school environment might have been a limitation because the home environment can also play an important role. Considering the limited amount of pocket money that students reported receiving and also the “gatekeeping” role of parents, adolescents' interaction with the food environment in and around the school was limited. Given this limited interaction with the food environment, adolescents' perceptions of the food environment could be a better proxy for their potential behavior. Furthermore, reducing our food environment measures to the number of food outlets might have simplified the complexity of the study. Measuring specific elements of the food environment that we identified as relevant for adolescents' purchasing behavior, such as availability, price and vicinity of fried food, could have been a better indicator. However, such a detailed assessment of the food environment was not feasible with the resources available. Despite this limitation, our detailed description of the food environment in and around the schools, the auditing of advertising by food group and by processing level, along with the diet characterization and perceptions of the adolescents, make this study uniquely important in light of the limited data on school food environments and adolescents' diet in Ethiopia, Africa and beyond.

### Conclusions and Implications

Our study found a high density of food outlets within the 0.5 km radius around the schools, as well as widespread promotion and display of ultra-processed foods and beverages in and around the schools. Such an environment is not conducive to promoting healthy dietary behaviors. While our study could not conclude that these environmental factors directly explain adolescents' diet or weight status, the influence of socio-economic and family backgrounds appeared more relevant.

These findings suggest that parents need to be directly involved in school interventions so that the home food environment can also be addressed. To ensure that adolescents make healthy dietary choices with their own pocket money, education on dietary quality through multiple channels is necessary. In addition, the currently widespread unhealthy choices in the school food environment need to be regulated. Advertising of unhealthy food and beverages in and around schools should be restricted and food and beverages offered in school cafeterias should follow food-based dietary guidelines (41), which are currently being drafted and validated in Ethiopia (42).

## Data Availability Statement

The raw data supporting the conclusions of this article will be made available by the authors, without undue reservation.

## Ethics Statement

The studies involving human participants were reviewed and approved by the study protocol was approved by the College of Natural and Computational Science Institutional Review Board of the Addis Ababa University (No. IRB/035/2018). Informed written consent was obtained from all study participants prior to data collection. For participants under 18 years, additional parental/guardian-informed written consent/ascent was obtained. All interviews were conducted on school premises and after school hours with no school staff present. Written informed consent to participate in this study was provided by the participants' legal guardian/next of kin.

## Author Contributions

UT conceptualized the research question, conducted the analyses, and authored the paper. KB coordinated the data collection. SE assisted in data collection and analysis. ET, EF, MH, MP, and KB assisted with conceptualizing the study, interpreting the results, and revising the manuscript. All authors contributed to the article and approved the submitted version.

## Funding

This work was undertaken as part of the CGIAR Research Program on Agriculture for Nutrition and Health (A4NH; https://a4nh.cgiar.org/our-research/flagship-1/).

## Author Disclaimer

The opinions expressed here belong to the authors, and do not necessarily reflect those of A4NH or CGIAR.

## Conflict of Interest

The authors declare that the research was conducted in the absence of any commercial or financial relationships that could be construed as a potential conflict of interest.

## Publisher's Note

All claims expressed in this article are solely those of the authors and do not necessarily represent those of their affiliated organizations, or those of the publisher, the editors and the reviewers. Any product that may be evaluated in this article, or claim that may be made by its manufacturer, is not guaranteed or endorsed by the publisher.
