# Supplementary material for: Factors Influencing Adolescents' Dietary Behaviors in the School and Home Environment in Addis Ababa, Ethiopia
Source: Front Public Health. 2022 Apr 8;10:861463. doi: 10.3389/fpubh.2022.861463 (PMC9024113; doi:10.3389/fpubh.2022.861463)
Supplement: Supplementary file 1 [file Data_Sheet_1.pdf]

Supplementary material 1 – Food outlet categories

1. Supermarket/small shop: Modern retail outlet with checkout counters, big selection of food items. Examples include supermarket chains called Bambis, Hirut or Fresh corner
2. Kiosk: usually tiny house/hut on the road, usually only one or two sellers. Not possible to enter. Selling sodas, snacks, sometimes eggs, matches, phone credit etc
3. Fruit and vegetable stall: mostly selling fruits and vegetables, sometimes also other small food items.
4. Open market: open space, with multiple stalls for different food items, such as Shola or Merkato.
5. Informal street seller mobile: has a cart with one or two different fruit or vegetable items. Not at the same place at regular times.
6. Informal street seller immobile: has a small space on the sidewalk with for instance onions, chili, tomatoes or candy. Not at the same place at regular times. Categorized into:
    a. Fruit or vegetable
    b. Candy
7. Bakery: only selling bread, rolls, sometimes cakes or even eggs.
8. Butcher: sells only beef meat
9. Kebele shop/cooperative: sells subsidized food items like oil, sugar, flour
10. Street food: sells different types of fried foods on the side of the road or as part of another shop
11. Café:
    a. Traditional/shybet: traditional Ethiopian coffee, also some Ethiopian food
    b. Modern/Kekbet: chains that sell cakes as well as fast food
12. Restaurant: serves hot meals, such as lunch and dinner
